# The Validation of the Italian Version of the Munich Swallowing Score (IT-MUCSS) Against the Fiberoptic Endoscopic Evaluation of Swallowing and Food Intake Modalities in Patients with Neurogenic Dysphagia: A Cross-Sectional Study

**DOI:** 10.3390/jcm14061942

**Published:** 2025-03-13

**Authors:** Giorgia Gottardo, Maria Zampieri, Maria Luisa Costanza, Marta Scamardella, Elena Castagnetti, Isabella Koch, Lorenza Maistrello, Sara Nordio

**Affiliations:** 1 IRCCS San Camillo Hospital, Via Alberoni 70, 30174 Venice, Italy; gottardo.giorgia@aou.mo.it (G.G.); maria.zampieri@hsancamillo.it (M.Z.); marialuisacostanza@hsancamillo.it (M.L.C.); marta.scamardella@asl5.liguria.it (M.S.); isabellalogo@gmail.com (I.K.); lorenza.maistrello@hsancamillo.it (L.M.); 2 Azienda Unità Sanitaria Locale-IRCCS di Reggio Emilia, Viale Murri 9, 42123 Reggio Emilia, Italy; elenacastagnetti98@gmail.com

**Keywords:** deglutition, functional oral intake, neurogenic dysphagia, validation, fiberoptic endoscopic evaluation of swallowing, tracheal tube

## Abstract

**Background/Objectives**: Oral intake and secretions need to be assessed separately, especially in patients with tracheal tubes, as they are vital for dysphagia treatment and may require different management strategies. This study aims to validate the Italian version of the Munich Swallowing Score (IT-MUCSS) by examining its content and construct validity in relation to the fiberoptic endoscopic evaluation of swallowing (FEES) and oral intake in adults with neurogenic dysphagia, as well as assessing intra- and inter-rater reliability. This tool is clinically and scientifically useful as it includes two subscales: IT-MUCSS-Saliva, which assesses saliva/secretion management and the presence of a tracheal tube, and IT-MUCSS-Alimentazione, which evaluates feeding methods. **Methods**: In this prospective cross-sectional study, a total of 50 dysphagic patients with a neurological diagnosis were recruited from a neuro-rehabilitation hospital and underwent both clinical and instrumental assessments. The main outcome measures included evaluating food and liquid intake using the Italian versions of the Functional Oral Intake Scale (FOIS-It) and the IT-MUCSS. Pharyngeal residues were assessed using the Yale Pharyngeal Residue Severity Rating Scale (IT-YPRSRS), and airway penetration/aspiration were evaluated using the Penetration–Aspiration Scale (PAS) during FEES. **Results**: The IT-MUCSS demonstrated excellent reproducibility (K = 0.91) and internal consistency (Cronbach’s alpha = 0.72). Strong correlations were found between IT-MUCSS and the FOIS-It scale, indicating the effective assessment of dysphagia. Test–retest reliability was high (ICC = 0.96 for total score). Construct validity was confirmed through significant correlations with instrumental measures during FEES. **Conclusions**: The IT-MUCSS is a valid tool for assessing functional oral intake and the management of saliva/secretions, specifically in relation to the level of saliva/secretions management compared to FEES measures of swallowing safety and efficiency in patients with neurogenic dysphagia.

## 1. Introduction

Oropharyngeal Dysphagia (OD) is a multifactorial clinical disorder affecting the swallowing of secretions, fluids and/or food, and can be caused by neurological diseases [1]. Prevalence rates reach 80% in the stroke population, 81% in patients with Parkinson’s disease (PD), 30% in those with traumatic brain injury (TBI), and 30–100% in individuals depending on their type of motor neuron disease (MND) and stage of disease [2,3]. This condition significantly impairs both swallowing safety and efficiency. Swallowing safety involves the ability to transfer the bolus from the mouth to the stomach without significant penetration or aspiration into the lower airways, while swallowing efficiency relates to the reduction in pharyngeal residues. In particular, swallowing safety refers to the protection of the airway during swallowing to prevent pulmonary aspiration. This involves two key elements: anatomical protection, which consists of the closure of the airway by the vocal cords and epiglottis, as well as the movement of the larynx to prevent food from entering the trachea, and coordination between swallowing and breathing, where swallowing is synchronized with breathing to minimize the risk of aspiration [4]. This typically occurs during a pause in the breathing cycle, when the airway is less likely to be compromised. On the other hand, swallowing efficiency refers to an individual’s ability to effectively and safely swallow food or liquids with minimal residue left in the oral cavity, pharynx, or larynx after swallowing [5]. It is typically measured by the amount of material that is successfully cleared from the mouth and throat, indicating how well the swallowing process is functioning [5]. High swallowing efficiency means that most of the bolus is swallowed without retention, thereby reducing the risk of aspiration or other complications. When these functions are impaired, as in the case of neurogenic dysphagia, respiratory problems such as aspiration pneumonia may occur [6,7,8], as well as malnutrition and dehydration, resulting in increased morbidity and mortality [1]. Furthermore, swallowing disorders can entail a prolonged length of hospital stay, long-term artificial nutrition, and an increased risk of institutionalization [9], as well as significantly reduce quality of life [10].

To date, the gold standards for the instrumental assessment of swallowing safety and efficiency are the Videofluoroscopic Swallowing Study (VFSS) [11] and Fiberoptic Endoscopic Evaluation of Swallowing (FEES) [12]. Results from instrumental evaluations should be considered along with those from clinical assessments [13,14,15], since establishing dysphagia severity addresses different measures and domains [16]. Therefore, an assessment of severity should describe the functional level of intake, including the need of different degrees of non-oral supplementation, dietary adaptations, compensatory strategies and/or the level of saliva and secretion management [17]. Notably, patients with severe dysphagia require non-oral feeding such as nasogastric tubes (NG tubes) or percutaneous endoscopic gastrostomy (PEG), which are essential devices to prevent aspiration and ensure adequate nutritional intake [18]. Another crucial aspect of patient care, particularly for individuals with swallowing disorders caused by neurological diseases, is the management of saliva and secretions [19]. These conditions can significantly impact the body’s ability to process and clear saliva and secretions. The accumulation of secretions leads to both sensory and motor impairments, including a reduced swallowing frequency, diminished laryngeal adductor reflex, impaired pharyngeal squeeze maneuver, and decreased peak expiratory flow. These issues increase the risk of aspiration, respiratory infections, and compromised nutritional intake [20]. Indeed, patients with severe dysfunctions of non-nutritive swallowing may need a cuffed tracheostomy tube to reduce the risk of aspiration [21]. The presence of a tube can have a significant psychological and social impact on a patient’s life as it affects human needs, such as respiration and communication [22]. Additionally, it may negatively influence the rehabilitation process [23,24]. For these reasons, it is important to address the issue of decannulation in the rehabilitation setting, as it not only facilitates improved swallowing function but also reduces the risk of respiratory infections and major complications associated with prolonged tube use [25]. Although decannulation is considered a crucial milestone in the rehabilitation process, standardized protocols for managing it are still lacking. However, there is substantial evidence supporting a multidisciplinary approach to the decannulation process [26]. For a safe decannulation, guidelines recommend that the patient should be able to clear the oral tract, demonstrate efficient coughing, have effective swallowing, and exhibit improved alertness [27,28]. Another important factor is the type of tracheal cannula used, which plays a significant role in both respiratory and swallowing function. A cuffed tracheal cannula, commonly used in patients requiring mechanical ventilation or in the early stages of neuro-rehabilitation, provides a secure airway and protects the patient from aspiration [29]. However, it may affect swallowing and secretion management by limiting the natural movement of the upper airway [30]. Conversely, uncuffed cannulas are often used in patients with less critical airway protection needs and may allow for more natural swallowing patterns [31]. The first step is the evaluation of secretions management during cuff deflation, as only minimal suctioning of secretion from above the cuff should be needed; then, the patient should be able to breathe spontaneously with stable and sufficient oxygen saturation during the tube occlusion test [26]; in the later stages, increased tolerance to deflation should be documented. Indeed, the process of cuff deflation is a critical step in the weaning process from a tracheal cannula, where the patient gradually transitions from a cuffed cannula to an uncuffed one. This transition is necessary for restoring normal swallowing function and facilitating secretion management, as it allows for the re-establishment of airway control and improves the patient’s ability to clear saliva and secretions and cough effectively [32]. In some cases, capping the cannula, which involves temporarily blocking the tube, is used as part of this weaning process to assess the patient’s ability to breathe through their natural airways [33]. Understanding the impact of cuffed and uncuffed cannulas, capping training, and the weaning process is crucial for clinicians when selecting appropriate interventions and monitoring patient outcomes. Moreover, oral intake and saliva/secretion swallowing deserve a specific and separated assessment from oral intake modalities, as they play a crucial role in dysphagia treatment and may require different management strategies. Therefore, it is essential to incorporate these key elements into a comprehensive assessment of patients with neurogenic dysphagia.

There are a variety of clinical scales measuring the functional level of oral intake of food and liquid in dysphagic patients [34,35]; conversely, the management of saliva/secretion has been scarcely considered in the literature to date. Indeed, only a few clinical scales include this ability, typically addressing it in just one or two of the several levels describing oral intake [36,37,38]. To our knowledge, the varying degrees of saliva/secretion swallowing ability, amount of expectoration, frequency of suctioning, and presence and type of tracheal tube are not taken into account, except for in the Munich Swallowing Score (MUCSS) [39].

The MUCSS is a clinical tool for assessing functional food and fluid intake and saliva/secretion management, which are considered separately but compared, as well as for documenting the number of expectorations, the presence of a tracheal tube, and cuff deflation status. The MUCSS was originally developed for German-speaking populations in a first pilot study [40], and its content validity and inter-rater reliability were evaluated as acceptable.

Subsequently, Bartolome and colleagues validated the scale in English and investigated criterion validity and sensitivity to change in the MUCSS, comparing it with scales that evaluate dysphagic symptoms and global measures of disability, and reported overall consistent results [39].

The importance of adopting internationally validated instruments for evaluating nutritional intake modalities and management of secretions is that it can facilitate the comparability of results across different countries. Therefore, the present study aims to assess the psychometric properties of the Italian version of the MUCSS (IT-MUCSS), describing its content validity and construct validity against FEES and oral intake in adult patients with neurogenic dysphagia, as well as inter- and intra-rater reliability. 

## 2. Materials and Methods

### 2.1. Study Design

This cross-sectional study aimed to verify the psychometric properties of IT-MUCSS. The validation process was conducted in two phases: first, the translation, followed by the assessment of its psychometric properties, including test–retest reliability, internal consistency, and construct validity. The methodology was guided by the COnsensus-based Standards for the selection of health status Measurement INstruments (COSMIN) checklist [41]. This approach guaranteed that the translated scale retained its reliability and validity within the Italian context [42]. 

The study was conducted in accordance with the Declaration of Helsinki [43] and received prior approval from the relevant Ethics Committee (Prot. 2022.03). All participants provided written informed consent.

### 2.2. Translation Process

After receiving authorization from the original authors of the MUCSS (Bartolome G., personal communication), the translation process followed these steps [44]:

(i) Two German Speech–Language Therapists (SLTs), experienced in dysphagia, independently produced two Italian translations.
(ii) These two versions were discussed and merged after reaching a consensus.
(iii) Next, an otolaryngologist and a SLT, both native Italian speakers with excellent German proficiency, conducted two back-translations of the agreed-upon Italian version.
(iv) The two blind back-translations were reviewed and compared with the original version.
(v) An expert committee (comprising 15 SLTs, 2 otolaryngologists, 2 physiotherapists and a neurologist, all experienced in dysphagia) reviewed the Italian version, concluding that 14 out of the 16 items on the scale were clear.

However, two items did not achieve 80% agreement on clarity among the evaluators. The main concerns involved the terminology and grammatical structure of the original scale. As a result, the syntaxes of Item 2 and Item 3 of the Italian version of the Munich Swallowing Score—Saliva (MUCSS-S) were modified for greater clarity. Raters reviewed the final version and deemed it clearer.

The Italian version of the scale is reported in Appendix A.

### 2.3. Psychometric Validation

The IT-MUCSS was analyzed for reproducibility, test–retest reliability, internal consistency, and construct validity with a group of patients with neurogenic dysphagia. 

### 2.4. Study Population

Fifty hospitalized patients were enrolled between 1 March 2022 and 31 July 2024 to undergo a clinical assessment and FEES examination. The inclusion criteria were as follows: (1) neurological etiology (stroke, PD, multiple sclerosis, amyotrophic lateral sclerosis, cerebrovascular infection, spinocerebellar ataxia, myasthenia gravis, anoxia, brain tumor, or traumatic brain injury); (2) diagnosed Oropharyngeal Dysphagia (OD) (by a neurologist); and (3) age over 18 years. Moreover, all the patients included in the study were from the Italian population and were fluent in the Italian language to ensure accurate assessment and communication during the evaluation. Exclusion criteria included the inability to maintain a stable sitting position during endoscopy or any contraindication to the FEES procedure, such as fever or agitation that compromised participation. 

### 2.5. Data Collection Procedures

Demographic, clinical, and instrumental data were recorded and stored anonymously in a Microsoft Excel 2016 worksheet, saved on a computer hard drive.

The enrolled patients underwent a dysphagia assessment, including a clinical/bedside examination. Patients’ oral intake was recorded using the Italian version of the Functional Oral Intake Scale (FOIS-It) [45] and the IT-MUCSS-Alimentazione (IT-MUCSS-A) subscale. Saliva and secretions management were recorded using the IT-MUCSS-Saliva (IT-MUCSS-S). FOIS-It and IT-MUCSS grading were performed blindly by two expert SLTs through direct patient observation. The same clinicians completed the scales again after three days for the retest.

Following the clinical assessment, the 50 patients underwent FEES examination conducted by an otorhinolaryngologist and an SLT using a flexible transnasal laryngoscope and the Tele Pack system (KARL STORZ SE & Co. KG, Tuttlingen, Germany). Oral secretions were first traced using two drops of methylene blue, administered via a syringe (Chemil srl, Padova, Italy) to the mid-posterior third of the tongue, and the patient was asked to swallow twice at their own pace. The exam then followed the International Dysphagia Diet Standardisation Initiative (IDDSI) framework [46], with the following bolus types:

(i) liquids (5–10–20 mL of blue-dyed water ×3 trials per volume; IDDSI 0; <50 mPa s at 50 s^−1^ and 300 s^−1^);
(ii) semisolids (5–10–20 mL of pudding ×3 trials per volume; IDDSI 4; 2583.3 ± 10.41 mPa s at 50 s^−1^ and 697.87 ± 7.84 mPa s at 300 s^−1^);
(iii) solids (half a Crich biscuit ×2 trials; IDDSI 7 Regular).

The boluses were administered by an SLT in the cited order, consistent across all patients. The protocol was modified if a particular consistency or volume was considered unsafe or if severe swallowing impairment was observed. 

### 2.6. Outcome Measures

#### 2.6.1. The Italian Version of Munich Swallowing Score

The IT-MUCSS consists of two subscales, each with 8 levels. The first subscale, the IT-MUCSS-S, assesses saliva/secretions management in relation to the presence and type of tracheal cannula. The need for a tracheostomy tube for respiratory issues without saliva/secretion swallowing disorders is not considered in this score. The second subscale, the IT-MUCSS-A, assesses the management of various food and liquid consistencies, including postures, parenteral/enteral nutrition, and compensatory strategies. A total score (IT-MUCSS-SA), ranging from 2 (best score) to 16 (worst score), can be obtained by summing the two subscale values.

IT-MUCSS scoring can be performed through clinical and/or instrumental swallowing evaluations; clinicians can also complete the scale using patient files and/or verified reports [39]. 

#### 2.6.2. The Italian Version of Functional Oral Intake Scale

The FOIS-It [45] is a 7-level ordinal scale that indicates the functional level of oral intake for food and liquids. Level 7 represents a full oral diet with no restrictions, levels 6 to 4 describe a full oral diet with restrictions, levels 3 to 2 represent a mixed oral and tube intake, and level 1 indicates total tube dependence.

### 2.7. Swallowing Safety and Efficiency

A SLT and an otolaryngologist, both experienced in dysphagia, evaluated the FEES examinations by consensus, using the following two tools:

(i) Swallowing safety was assessed using the Penetration–Aspiration Scale (PAS) [47], an ordinal scale ranging from 1 to 8. Score 1 represents no penetration and aspiration, score 2 reflects transient penetration with ejection, scores 3 to 5 indicate penetration without ejection or reaching the vocal folds, and scores 6 to 8 indicate aspiration. PAS scores of 1 to 2 were considered to reflect normal swallowing function, as reported in studies assessing the PAS’s psychometric properties [48,49].
(ii) Swallowing efficiency was measured using the Italian version of the Yale Pharyngeal Residue Severity Rating Scale (IT-YPRSRS) [50]. This ordinal scale provides scores based on the amount and location (epiglottic vallecula and pyriform sinuses) of post-swallow residue, ranging from 1 (no residue) to 5 (severe residue). In this study, an IT-YPRSRS score greater than 3 was considered suggestive of clinically significant residue [51].

For each food consistency tested, the worst PAS and IT-YPRSRS scores from the three trials were used.

### 2.8. Statistical Analysis

Sample characteristics were described using basic descriptive statistics (i.e., mean, standard deviation, absolute frequencies, and percentages) for the demographic and clinical characteristics of patients.

After translation and adaptation, the new version retained both item-level features, such as internal consistency, and score-level features, such as reliability and construct validity.

Inter-rater reliability was assessed using Cohen’s K index [52,53,54]. The K coefficient ranges from 0 to 1, with K > 0.75 indicating excellent inter-rater reliability, 0.40 to 0.75 indicating fair to good reliability, and K < 0.40 indicating poor reproducibility [55,56].

Internal consistency was measured using Cronbach’s alpha index. Values of alpha greater than 0.70 were considered acceptable [42,57].

Test–retest reliability was assessed using the intraclass correlation coefficient (ICC) [58], with ICC > 0.85 indicating excellent reliability and ICC values between 0.70 and 0.85 indicating good reliability [59].

Spearman correlation tests between the IT-MUCSS scale, clinical (FOIS-It), and fibro-endoscopic measures (i.e., PAS and IT-YPRSRS) were performed to evaluate construct validity. Correlation was considered perfect if rho > 0.9, strong if between 0.7 and 0.9, moderate if between 0.4 and 0.6, weak if between 0.1 and 0.3, and no correlation if rho = 0 [60]. 

Statistical analysis was performed using R Studio software v. 4.2.3. *p* values of <0.05 were considered significant.

## 3. Results

### 3.1. Sample Characteristics

Table 1 shows the results for the main summary statistics carried out on the sample. Of the 50 patients tested, 21 were females (42%) and 29 were males (58%), and the sample had a mean age of 67 years. The mean time since diagnosis was 18 months. At baseline, 64% of patients (n = 32) did not have a tracheal tube and 50% (n = 25) were on enteral nutrition.

### 3.2. Psychometric Reliability and Validity

#### 3.2.1. Reproducibility: Concordance Between Judges

The Cohen’s K-index values assessed by the two raters indicated excellent reproducibility for both the IT-MUCSS-S domain (K_S_ = 0.89) and the IT-MUCSS-A domain (K_N_ = 0.88), with an overall agreement of 92% for the first domain and 90% for the second. For the overall scale score (IT-MUCCS-SA), the percentage of agreement was 92% (K = 0.91).

#### 3.2.2. Internal Consistency

The internal consistency of the scale was found to be acceptable, with a Cronbach’s alpha value of 0.72.

### 3.3. Test–Retest

Test–retest reliability was assessed using the intraclass correlation coefficient (ICC). The results indicate excellent reliability for both the total scale score (ICC = 0.96) and the separate IT-MUCSS-S (ICC = 0.97) and IT-MUCSS-A domains (ICC = 0.96). 

### 3.4. Construct Validity

Spearman correlation tests were conducted between the IT-MUCSS domains (both separately and as a total scale score), clinical measures (i.e., FOIS-It), and the instrumental measures applied during FEES (i.e., PAS and IT-YPRSRS) to assess construct validity.

Highly significant moderate-to-very-strong correlations were found between the clinical assessment of dysphagia using the FOIS-It scale, swallowing safety, and swallowing efficiency for each consistency examined (as measured by PAS and the IT-YPRSRS during FEES) and all the components of the IT-MUCSS scale (Table 2).

Specifically, a moderate negative correlation was found between FOIS-It and the IT-MUCSS-S, while a strong negative correlation was observed between FOIS-It and both the IT-MUCSS-A and IT-MUCSS-SA.

High positive correlations were found with a high level of significance between the IT-MUCSS-S, IT-MUCSS-N, IT-MUCSS-SA, and the FEES scale scores. The strongest correlations emerged when comparing the IT-MUCSS-A and the degree of penetration/aspiration measured by PAS (PAS—Solids, PAS—Semisolids, PAS—Liquids). Regarding pharyngeal saliva residues, strong positive correlations were found among all domains of the IT-MUCSS scale and both the IT-YPRSRS Pyriform Sinuses and IT-YPRSRS Vallecula. 

As all correlations were verified, construct validity was confirmed.

## 4. Discussions

The evaluation of food and liquid intake and saliva/secretion handling ability is of paramount importance in the management of patients with neurogenic dysphagia [34,61].

Among the different clinical scales that accompany the instrumental assessment of swallowing disorders, a quick and reliable tool is needed to consider both of these aspects. The Italian guidelines for the management of patients with dysphagia report that the assessment and treatment of people with swallowing disorders vary in clinical practice due to a lack of standardized protocols and procedures [17]. This is especially true for dysphagic patients with tracheal tubes. The use of shared and valid scales that also consider the presence of tracheal tubes along with nutritional modalities would represent a first step toward achieving greater uniformity in both the scientific field and in clinical practice, thereby providing more valid evaluations of treatment outcomes. Therefore, the aim of this study was to test the psychometric properties of IT-MUCSS. First, the scale has been appropriately translated and culturally adapted for the Italian population to ensure its validity, in accordance with the guidelines established by Sousa et al. [44]. Then, in this study, we contribute to verifying the inter-rater reliability and internal consistency of the MUCSS-IT, ensuring the scale’s robustness and coherence among its components, as the scale has preserved its accuracy and reliability. These results are consistent with those of the initial validation study [30], further reinforcing the reliability of the MUCSS-IT for clinical use across diverse settings and with different operators. Nevertheless, the scientific process of validating outcome measures is regulated by the COSMIN [41] framework, which involves several steps, including the assessment of reliability regarding both test–retest and inter-rater reliability. The COSMIN framework was partially applied for the validation of the original version of the MUCSS, as test–retest measurement has not been verified [39,40].

To our knowledge, this study is the first in the literature to analyze the test–retest reliability of the MUCSS-IT, providing a significant contribution to its psychometric validation. In fact, the ICC values demonstrate the stability of individual measures over time. Ultimately, we aimed to demonstrate the construct validity of the IT-MUCSS against a gold-standard instrumental examination of swallowing (FEES) in adult patients with OD due to neurological etiology. 

Consistent with previous results [39], the present study supports the convergent validity hypothesis that functional oral intake and saliva/secretions management in relation to the presence and type of tracheal tube are associated with swallowing safety assessed using PAS. In fact, there is a direct correlation between the IT-MUCSS-S and aspiration severity of secretions (traced with methylene blue during FEES). A strong correlation between the IT-MUCSS-A, IT-MUCSS-SA, and swallowing safety for all consistencies was also found.

Regarding swallowing efficiency, aligned with the previous study [39], the IT-YPRSRS was found to correlate with the IT-MUCSS-A and IT-MUCSS-SA for solids, semisolids, and liquids, and the results from secretions evaluation suggest a good correlation with the IT-MUCSS-S as well. These findings are partially consistent with the previous research on the MUCSS, which is associated with PAS and YPRSRS total results, as Bartolome et al. did not apply statistical analysis differentiating between textures. However, the impact of liquid consistency and food texture on swallowing behavior both in healthy individuals [62] and patients with OD is well known [48]. Indeed, the properties of food and liquids may modify swallow measures such as penetration/aspiration, oral transit time, lingual pressures, submental muscle contraction, oral and pharyngeal residue, hyoid and laryngeal movement, pharyngeal and upper esophageal sphincter pressures, and total swallow duration [63]. Given this fact, it is recommended to conduct analyses differentiating between consistencies.

Ultimately, the concurrent validity analysis through the FOIS-It demonstrates significant correlations with the IT-MUCSS-A and IT-MUCSS-SA, underscoring their validity in outlining a functional oral intake profile for patients with neurogenic dysphagia. In contrast, the IT-MUCSS-S shows a low correlation with FOIS-It. This result can be discussed in light of previous studies that found a dissociation between functional oral intake and management of secretions [64,65]. Furthermore, it is important to note that frequently in neurogenic dysphagia the level of functional oral intake may depend not only on swallowing safety and efficiency but also on other relevant factors, such as the presence of cognitive and/or behavioral deficits [66], performance during an entire meal [67], possible assistance by caregivers, and the use of strategies to address swallowing difficulties. Considering all these aspects, we may find contrasting results between the performance at FEES and clinical scales.

In addition, Bartolome et al. [39] found that the MUCSS reflected positive changes in both the status of saliva/secretion swallowing and oral food intake in a group of patients with neurogenic dysphagia. In the present study, we extended IT-MUCSS validation to a small sample of patients affected by neurodegenerative diseases. All the IT-MUCSS levels were represented within the recruited sample, highlighting the adequate applicability of the scale to OD from conditions other than stroke. Moreover, this population was suitable for an evaluation that considered changes in saliva/secretion management [68,69] and the presence and type of tracheal tube, allowing for the monitoring of changes over time [69]. 

### Limitations

Finally, some limitations of this study need to be reported. First, the sample was recruited in a neuro-rehabilitation hospital and was composed of patients with OD due to various etiologies, mostly stroke, none of whom were in acute condition. Future studies should validate IT-MUCSS with a larger sample size and by collecting sub-samples homogeneous for diagnosis and time of onset. Secondly, three different consistencies were used in this study, representing only a portion of the boluses available for FEES [37]. Furthermore, the evaluation of secretion management was performed by tracing oral secretions with methylene blue and applying the IT-YPRSRS, which is validated for boluses of different consistencies. However, for future studies, it would be appropriate to use a standardized procedure specifically designed for the assessment of secretions.

## 5. Conclusions

The IT-MUCSS demonstrated good internal consistency, test–retest reliability, and inter-rater reliability, confirming its appropriateness and potential inclusion in the Italian clinical and research context.

It is a valid tool for assessing functional oral intake and saliva/secretions management concerning the presence of a tracheal tube, and reflecting swallowing safety and efficiency as analyzed during FEES in adult patients with OD from various neurological conditions.

## Figures and Tables

**Table 1:** Sample characteristics.

Participants (N = 50)	
Age, Years, Mean ± SD	67 ± 12
Sex, Female/Male, n (%)	21 (42%)/29 (58%)
Diagnosis, n (%)	
Hemorrhagic Stroke	14 (28%)
Ischaemic Stroke	17 (34%)
Brain Tumor	2 (4%)
Parkinson Disease	3 (6%)
Amyotrophic Lateral Sclerosis	4 (8%)
Multiple Sclerosis	3 (6%)
Traumatic Brain Injury	4 (8%)
Others	3 (6%)
Time Of Onset, Months, Mean ± SD	18 ± 48
Tracheal Tube, Absence/Presence/na, n (%)	32 (64%)/18 (36%)
Enteral Nutrition, Absence/Presence, n (%)	25 (50%)/25 (50%)

Values are expressed as mean ± standard deviation (SD) for quantitative variables and absolute frequency (n) and percentage frequency (%) for qualitative variables.

**Table 2:** Spearman’s rank correlation coefficients between IT-MUCSS scales and FOIS-It, PAS, and IT-YPRSRS for secretions and each consistency type.

(N = 50)	IT-MUCSS-S	IT-MUCSS-A	IT-MUCSS-SA
FOIS-It	−0.44 *	−0.91 *	−0.82 *
PAS—Solids (IDDSI 7)	-	0.94 *	0.86 *
PAS—Semisolids (IDDSI 4)	-	0.95 *	0.83 *
PAS—Liquids (IDDSI 0)	-	0.91 *	0.83 *
PAS—Secretions	0.83 *	-	-
IT-YPRSRS PS—Solids (IDDSI 7)	-	0.73 *	0.76 *
IT-YPRSRS PS—Semisolids (IDDSI 4)	-	0.86 *	0.78 *
IT-YPRSRS PS—Liquids (IDDSI 0)	-	0.86 *	0.81 *
IT-YPRSRS PS—Secretions	0.81 *	-	-
IT-YPRSRS V—Solids (IDDSI 7)	-	0.72 *	0.73 *
IT-YPRSRS V—Semisolids (IDDSI 4)	-	0.89 *	0.80 *
IT-YPRSRS V—Liquids (IDDSI 0)	-	0.89 *	0.82 *
IT-YPRSRS V—Secretions	0.79 *	-	-

Significant Spearman correlation indices indicated by * *p* < 0.05. IT-YPRSRS PS = IT-YPRSRS Pyriform Sinuses. IT-YPRSRS V = IT-YPRSRS Valleculae.

## Data Availability

The datasets generated or analyzed during the current study are available from the corresponding author on reasonable request.

## Abbreviations

The following abbreviations are used in this manuscript:

OD	Oropharyngeal Dysphagia
PD	Parkinson’s Disease
TBI	Traumatic brain injury
MDN	Motor neuron disease
VFSS	Videofluoroscopic Swallowing Study
FEES	Fiberoptic Endoscopic Evaluation of Swallowing
NG tube	Nasogastric tube
PEG	Percutaneous endoscopic gastrostomy
MUCCS	Munich Swallowing Score
COSMIN	COnsensus-based Standards for the selection of health status Measurement INstruments
SLT	Speech and Language Therapist
IDDSI	International Dysphagia Diet Standardisation Initiative
FOIS	Functional Oral Intake Scale
PAS	Penetration–Aspiration Scale
IT-YPRSRS	Italian version of the Yale Pharyngeal Residue Severity Rating Scale
ICC	Intraclass correlation coefficient

## Appendix A

**Munich Swallowing Score—Saliva (MUCSS-S)**	
1	Cannula tracheostomica assente, deglutizione della saliva non compromessa
2	Cannula tracheostomica assente, occasionalmente voce gorgogliante e/o espettorazione (intervalli > 1 ora)
3	Cannula tracheostomica assente, voce gorgogliante e/o espettorazione frequenti (intervalli ≤ 1 ora)
4	Cannula tracheostomica senza cuffia, cannula fenestrata o *stoma stent* per aspirazione di saliva/secrezioni
5	Cannula tracheostomica tenuta scuffiata per > 12 ore e < 24 ore al giorno
6	Cannula tracheostomica tenuta scuffiata per > 1 ora e ≤ 12 ore al giorno
7	Cannula tracheostomica tenuta scuffiata per ≤ 1 ora al giorno
8	Cannula tracheostomica sempre cuffiata

**Munich Swallowing Score—Alimentazione (MUCSS-A)**	
1	Alimentazione orale totale senza restrizioni
2	Alimentazione orale totale con lievi restrizioni: diverse consistenze di alimenti e almeno una consistenza liquida senza l’uso di strategie di compenso oppure uso di strategie di compenso senza restrizioni di consistenze di alimenti e di liquidi
3	Alimentazione orale totale con moderate restrizioni: diverse consistenze di alimenti e almeno una consistenza liquida con uso di strategie di compenso
4	Alimentazione orale totale con severe restrizioni: solo una consistenza di alimenti e/o liquidi addensati con o senza strategie di compenso
5	Alimentazione prevalentemente per via orale: più della metà del fabbisogno giornaliero, fabbisogno residuo per via enterale/parenterale
6	Alimentazione orale parziale: >10 cucchiaini al giorno fino alla metà del fabbisogno giornaliero, fabbisogno residuo per via enterale/parenterale
7	Minima assunzione di cibo per via orale: ≤10 cucchiaini al giorno, fabbisogno residuo per via enterale/parenterale
8	Assunzione di cibo esclusivamente per via enterale/parenterale
	Punteggio MUCCS-S: ____. Punteggio MUCCS-A: ____. Punteggio totale (sommare punteggio MUCCS-S e MUCSS-A): ________.
	Punteggio totale: 2 (assenza di disfagia); 16 (grave disfagia).

## References

[1] Banda K.J., Chu H., Chen R., Kang X.L., Jen H.-J., Liu D., Shen Hsiao S.-T., Chou K.-R. (2022). Prevalence of Oropharyngeal Dysphagia and Risk of Pneumonia, Malnutrition, and Mortality in Adults Aged 60 Years and Older: A Meta-Analysis. Gerontology.

[2] Guan X.-L., Wang H., Huang H.-S., Meng L. (2015). Prevalence of Dysphagia in Multiple Sclerosis: A Systematic Review and Meta-Analysis. Neurol. Sci..

[3] Panebianco M., Marchese-Ragona R., Masiero S., Restivo D.A. (2020). Dysphagia in Neurological Diseases: A Literature Review. Neurol. Sci..

[4] Ouahchi Y., Ben Salah N., Mjid M., Hedhli A., Abdelhedi N., Beji M., Toujani S., Verin E. (2019). Breathing pattern during sequential swallowing in healthy adult humans. J. Appl. Physiol..

[5] Jardine M., Miles A., Allen J., Leonard R. (2020). Quantifying post-swallow residue in healthy aging. Perspect. ASHA Spec. Interest Groups.

[6] Rofes L., Arreola V., Almirall J., Cabré M., Campins L., García-Peris P., Speyer R., Clavé P. (2011). Diagnosis and Management of Oropharyngeal Dysphagia and Its Nutritional and Respiratory Complications in the Elderly. Gastroenterol. Res. Pract..

[7] Feng M.-C., Lin Y.-C., Chang Y.-H., Chen C.-H., Chiang H.-C., Huang L.-C., Yang Y.-H., Hung C.-H. (2019). The Mortality and the Risk of Aspiration Pneumonia Related with Dysphagia in Stroke Patients. J. Stroke Cerebrovasc. Dis..

[8] Eltringham S.A., Kilner K., Gee M., Sage K., Bray B.D., Pownall S., Smith C.J. (2018). Impact of Dysphagia Assessment and Management on Risk of Stroke-Associated Pneumonia: A Systematic Review. Cerebrovasc. Dis..

[9] Leira J., Maseda A., Lorenzo-López L., Cibeira N., López-López R., Lodeiro L., Millán-Calenti J.C. (2023). Dysphagia and Its Association with Other Health-Related Risk Factors in Institutionalized Older People: A Systematic Review. Arch. Gerontol. Geriatr..

[10] Jones E., Speyer R., Kertscher B., Denman D., Swan K., Cordier R. (2018). Health-related quality of life and oropharyngeal dysphagia: A systematic review. Dysphagia.

[11] Costa M.M.B. (2010). Videofluoroscopy: The Gold Standard Exam for Studying Swallowing and Its Dysfunction. Arq. Gastroenterol..

[12] Langmore S.E. (2017). History of Fiberoptic Endoscopic Evaluation of Swallowing for Evaluation and Management of Pharyngeal Dysphagia: Changes over the Years. Dysphagia.

[13] Warnecke T., Labeit B., Schroeder J., Reckels A., Ahring S., Lapa S., Claus I., Muhle P., Suntrup-Krueger S., Dziewas R. (2021). Neurogenic Dysphagia: Systematic Review and Proposal of a Classification System. Neurology.

[14] Labeit B., Michou E., Hamdy S., Trapl-Grundschober M., Suntrup-Krueger S., Muhle P., Bath P.M., Dziewas R. (2023). The Assessment of Dysphagia after Stroke: State of the Art and Future Directions. Lancet Neurol..

[15] Garand K.L., McCullough G., Crary M., Arvedson J.C., Dodrill P. (2020). Assessment Across the Life Span: The Clinical Swallow Evaluation. Am. J. Speech Lang. Pathol..

[16] Nordio S., Di Stadio A., Koch I., Stritoni P., Meneghello F., Palmer K. (2020). The Correlation between Pharyngeal Residue, Penetration/Aspiration and Nutritional Modality: A Cross-Sectional Study in Patients with Neurogenic Dysphagia. Acta Otorhinolaryngol. Ital..

[17] Speyer R., Cordier R., Farneti D., Nascimento W., Pilz W., Verin E., Walshe M., Woisard V. (2022). White Paper by the European Society for Swallowing Disorders: Screening and Non-Instrumental Assessment for Dysphagia in Adults. Dysphagia.

[18] Gomes C.A., Andriolo R.B., Bennett C., Lustosa S.A., Matos D., Waisberg D.R., Waisberg J. (2015). Percutaneous Endoscopic Gastrostomy versus Nasogastric Tube Feeding for Adults with Swallowing Disturbances. Cochrane Database Syst. Rev..

[19] McGeachan A.J., Mcdermott C.J. (2017). Management of oral secretions in neurological disease. Pract. Neurol..

[20] Hunting A., Steffanoni B., Jacques A., Miles A. (2023). Accumulated secretions and Associated Aerodigestive function in patients with Dysphagia. Am. J. Speech-Lang. Pathol..

[21] Bailey R.L. (2005). Tracheostomy and Dysphagia: A Complex Association. Perspect. Swallowing Swallowing Disord. Dysphagia.

[22] Nakarada-Kordic I., Patterson N., Wrapson J., Reay S.D. (2018). A Systematic Review of Patient and Caregiver Experiences with a Tracheostomy. Patient Patient-Centered Outcomes Res..

[23] Keren O., Cohen M., Lazar-Zweker I., Groswasser Z. (2001). Tracheotomy in Severe TBI Patients: Sequelae and Relation to Vocational Outcome. Brain Inj..

[24] Enrichi C., Battel I., Zanetti C., Koch I., Ventura L., Palmer K., Meneghello F., Piccione F., Rossi S., Lazzeri M. (2017). Clinical Criteria for Tracheostomy Decannulation in Subjects with Acquired Brain Injury. Respir. Care.

[25] Law J.H., Barnhart K., Rowlett W., De La Rocha O., Lowenberg S. (1993). Increased Frequency of Obstructive Airway Abnormalities with Long-Term Tracheostomy. Chest.

[26] Frank U., Mäder M., Sticher H. (2007). Dysphagic Patients with Tracheotomies: A Multidisciplinary Approach to Treatment and Decannulation Management. Dysphagia.

[27] Kutsukutsa J., Mashamba-Thompson T.P., Saman Y. (2017). Tracheostomy Decannulation Methods and Procedures in Adults: A Systematic Scoping Review Protocol. Syst. Rev..

[28] Kutsukutsa J., Kuupiel D., Monori-Kiss A., Del Rey-Puech P., Mashamba-Thompson T.P. (2019). Tracheostomy Decannulation Methods and Procedures for Assessing Readiness for Decannulation in Adults: A Systematic Scoping Review. Int. J. Evid. Based Healthc..

[29] Pandian V., Boisen S., Mathews S., Brenner M.J. (2019). Speech and safety in tracheostomy patients receiving mechanical ventilation: A systematic review. Am. J. Crit. Care.

[30] Ding R., Logemann J.A. (2005). Swallow physiology in patients with trach cuff inflated or deflated: A retrospective study. Head Neck.

[31] Bach J.R., Gonçalves M.R., Rodriguez P.L., Saporito L., Soares L. (2014). Cuff deflation: Rehabilitation in critical care. Am. J. Phys. Med. Rehabil..

[32] Zhou T., Wang J., Zhang C., Zhang B., Guo H., Yang B., Li Q., Ge J., Li Y., Niu G. (2022). Tracheostomy decannulation protocol in patients with prolonged tracheostomy referred to a rehabilitation hospital: A prospective cohort study. J. Intensive Care.

[33] Kim Y.K., Lee S.H., Lee J.W. (2017). Effects of Capping of the Tracheostomy Tube in Stroke Patients with Dysphagia. Ann. Rehabil. Med..

[34] Crary M.A., Mann G.D.C., Groher M.E. (2005). Initial Psychometric Assessment of a Functional Oral Intake Scale for Dysphagia in Stroke Patients. Arch. Phys. Med. Rehabil..

[35] Dungan S., Gregorio D., Abrahams T., Harrison B., Abrahams J., Brocato D., Davis C., Espana E., Garcia R., Smith S. (2019). Comparative Validity of the American Speech-Language-Hearing Association’s National Outcomes Measurement System, Functional Oral Intake Scale, and G-Codes to Mann Assessment of Swallowing Ability Scores for Dysphagia. Am. J. Speech Lang. Pathol..

[36] Warnecke T., Ritter M.A., Kröger B., Oelenberg S., Teismann I., Heuschmann P.U., Ringelstein E.B., Nabavi D.G., Dziewas R. (2009). Fiberoptic Endoscopic Dysphagia Severity Scale Predicts Outcome after Acute Stroke. Cerebrovasc. Dis..

[37] Nishimura K., Kagaya H., Shibata S., Onogi K., Inamoto Y., Ota K., Miki T., Tamura S., Saitoh E. (2015). Accuracy of Dysphagia Severity Scale Rating without Using Videoendoscopic Evaluation of Swallowing. Jpn. J. Compr. Rehabil. Sci..

[38] Sheppard J.J., Hochman R., Baer C. (2014). The Dysphagia Disorder Survey: Validation of an Assessment for Swallowing and Feeding Function in Developmental Disability. Res. Dev. Disabil..

[39] Bartolome G., Starrost U., Schröter-Morasch H., Schilling B., Fischbacher L., Kues L., Graf S., Ziegler W. (2021). Validation of the Munich Swallowing Score (MUCSS) in Patients with Neurogenic Dysphagia: A Preliminary Study. NeuroRehabilitation.

[40] Starrost U., Bartolome G., Schröter-Morasch H., Ziegler W., Fussenegger C., Marano C., Schilling B. (2012). Der Bogenhausener Dysphagiescore–BODS: Inhaltsvalidität Und Reliabilität. DysphagieForum.

[41] Mokkink L.B., Prinsen C.A.C., Bouter L.M., Vet H.C.W.D., Terwee C.B. (2016). The COnsensus-Based Standards for the Selection of Health Measurement INstruments (COSMIN) and How to Select an Outcome Measurement Instrument. Braz. J. Phys. Ther..

[42] Mokkink L.B., Terwee C.B., Patrick D.L., Alonso J., Stratford P.W., Knol D.L., Bouter L.M., De Vet H.C.W. (2010). The COSMIN Study Reached International Consensus on Taxonomy, Terminology, and Definitions of Measurement Properties for Health-Related Patient-Reported Outcomes. J. Clin. Epidemiol..

[43] World Medical Association (2013). Declaration of Helsinki: Ethical Principles for Medical Research Involving Human Subjects. JAMA.

[44] Sousa V.D., Rojjanasrirat W. (2011). Translation, Adaptation and Validation of Instruments or Scales for Use in Cross-cultural Health Care Research: A Clear and User-friendly Guideline. J. Eval. Clin. Pract..

[45] Ninfa A., Pizzorni N., Eplite A., Moltisanti C., Schindler A. (2022). Validation of the Italian Version of the Functional Oral Intake Scale (FOIS-It) Against Fiberoptic Endoscopic Evaluation of Swallowing and Nutritional Status. Dysphagia.

[46] Cichero J.A.Y., Lam P.T.L., Chen J., Dantas R.O., Duivestein J., Hanson B., Kayashita J., Pillay M., Riquelme L.F., Steele C.M. (2020). Release of Updated International Dysphagia Diet Standardisation Initiative Framework (IDDSI 2.0). J. Texture Stud..

[47] Colodny N. (2002). Interjudge and Intrajudge Reliabilities in Fiberoptic Endoscopic Evaluation of Swallowing (Fees^®^) Using the Penetration-Aspiration Scale: A Replication Study. Dysphagia.

[48] Steele C.M., Alsanei W.A., Ayanikalath S., Barbon C.E.A., Chen J., Cichero J.A.Y., Coutts K., Dantas R.O., Duivestein J., Giosa L. (2015). The Influence of Food Texture and Liquid Consistency Modification on Swallowing Physiology and Function: A Systematic Review. Dysphagia.

[49] Borders J.C., Brates D. (2020). Use of the Penetration-Aspiration Scale in Dysphagia Research: A Systematic Review. Dysphagia.

[50] Nordio S., Maistrello L., D’Imperio D., Favaretto N., Dellai A., Montino S., Agostinelli A., Ramacciotti G., Gheller F., Berta G. (2023). Validity and Reliability of the Italian Translation of the Yale Pharyngeal Residue Severity Rating Scale. Acta Otorhinolaryngol. Ital..

[51] Kelly A.M., Macfarlane K., Ghufoor K., Drinnan M.J., Lew-Gor S. (2008). Pharyngeal Residue across the Lifespan: A First Look at What’s Normal. Clin. Otolaryngol..

[52] Cohen J. (1960). A Coefficient of Agreement for Nominal Scales. Educ. Psychol. Meas..

[53] George D., Mallery P. (2019). IBM SPSS Statistics 26 Step by Step: A Simple Guide and Reference.

[54] Gisev N., Bell J.S., Chen T.F. (2013). Interrater Agreement and Interrater Reliability: Key Concepts, Approaches, and Applications. Res. Soc. Adm. Pharm..

[55] Fleiss J.L. (1971). Measuring Nominal Scale Agreement among Many Raters. Psychol. Bull..

[56] Gwet K.L. (2014). Handbook of Inter-Rater Reliability: The Definitive Guide to Measuring the Extent of Agreement among Raters.

[57] Nunnally J. (1978). Psychometric Theory.

[58] Deyo R.A., Diehr P., Patrick D.L. (1991). Reproducibility and Responsiveness of Health Status Measures Statistics and Strategies for Evaluation. Control. Clin. Trials.

[59] Atkinson G., Nevill A.M. (1998). Statistical Methods For Assessing Measurement Error (Reliability) in Variables Relevant to Sports Medicine. Sports Med..

[60] Dancey C. (2007). Statistics Without Maths for Psychology.

[61] Donzelli J., Wesling M., Brady S., Craney M. (2003). Predictive Value of Accumulated Oropharyngeal Secretions for Aspiration during Video Nasal Endoscopic Evaluation of the Swallow. Ann. Otol. Rhinol. Laryngol..

[62] Peña-Chávez R.E., Schaen-Heacock N.E., Hitchcock M.E., Kurosu A., Suzuki R., Hartel R.W., Ciucci M.R., Rogus-Pulia N.M. (2023). Effects of Food and Liquid Properties on Swallowing Physiology and Function in Adults. Dysphagia.

[63] Newman R., Vilardell N., Clavé P., Speyer R. (2016). Effect of Bolus Viscosity on the Safety and Efficacy of Swallowing and the Kinematics of the Swallow Response in Patients with Oropharyngeal Dysphagia: White Paper by the European Society for Swallowing Disorders (ESSD). Dysphagia.

[64] Ledl C., Mertl-Rötzer M. (2017). The Impact of Various Consistencies on Swallowing Safety in Neurogenic Dysphagia. Dysphagia.

[65] Sörös P., Inamoto Y., Martin R.E. (2009). Functional Brain Imaging of Swallowing: An Activation Likelihood Estimation Meta-analysis. Hum. Brain Mapp..

[66] Jo S.Y., Hwang J.-W., Pyun S.-B. (2017). Relationship Between Cognitive Function and Dysphagia After Stroke. Ann. Rehabil. Med..

[67] Brates D., Molfenter S. (2021). The Influence of Age, Eating a Meal, and Systematic Fatigue on Swallowing and Mealtime Parameters. Dysphagia.

[68] Britton D., Karam C., Schindler J.S. (2018). Swallowing and Secretion Management in Neuromuscular Disease. Clin. Chest Med..

[69] Wu J., Burdick R., Deckelman C., Gustafson S., Yee J., Rogus-Pulia N. (2023). Management of Dysphagia in Neurodegenerative Disease. Curr. Otorhinolaryngol. Rep..

